# The correlation between muscle loss and the severity of vascular stenosis in elderly patients with peripheral artery disease: a retrospective analysis utilizing computed tomography

**DOI:** 10.1007/s40520-025-02996-8

**Published:** 2025-03-11

**Authors:** Yanyang Zhang, Wenxin Zhao, Zuoguan Chen, Yixuan Wang, Xihao Zhang, Xue Chang, Yongjun Li, Jihong Yang

**Affiliations:** 1 Department of Geriatrics, Beijing Hospital, National Center of Gerontology, Institute of Geriatric Medicine, Chinese Academy of Medical Sciences & Peking Union Medical College, Beijing, 100005 China; 2 Graduate School of Chinese Academy of Medical Sciences & Peking Union Medical College, Beijing, 100005 China; 3 Department of Vascular Surgery, Beijing Hospital, National Center of Gerontology, Institute of Geriatric Medicine, Chinese Academy of Medical Sciences & Peking Union Medical College, Beijing, 100005 China; 4 Department of Imaging, Beijing Hospital, National Center of Gerontology, Institute of Geriatric Medicine, Chinese Academy of Medical Sciences & Peking Union Medical College, Beijing, 100005 China; 5 Department of Geriatric, Beijing United Family Hospital, Beijing, 100015 China

**Keywords:** Peripheral artery disease, Muscle loss, Vascular stenosis, Computed tomography, Sarcopenia

## Abstract

**Background:**

Peripheral artery disease (PAD) is a globally prevalent atherosclerotic disease associated with an increased risk of cardiovascular and cerebrovascular diseases and a poor prognosis. Skeletal muscle loss (sarcopenia) is particularly common in patients with PAD and is closely associated with poor prognosis.

**Aims:**

The aim of this study was to evaluate the area, density and fat infiltration of skeletal muscle in patients with PAD by CT, and to analyze their relationship with the degree of vascular stenosis.

**Methods:**

A total of 233 PAD patients who underwent lower extremity CTA in Beijing Hospital were included in this study. Image segmentation was performed using Slice-O-Matic® software, and parameters such as skeletal muscle area, density, and fat infiltration were measured at L3, L4, mid-thigh, and maximum soft tissue cross section of the lower leg. At the same time, the degree of lower extremity arterial stenosis was evaluated by CTA. The lower extremity arterial stenosis severity was graded as 0 (0–30%), 1 (31–50%), 2 (51–70%), 3 (71–99%), or 4 (occlusion).Then the CTA-score was calculated by summing the stenosis scores of the abdominal aorta and the lower limb arteries.

**Results:**

Patients were categorized into high (n = 113) and low (n = 120) CTA score groups. Among males, those in the low score group had higher muscle indices at L3, though not statistically significant. However, thigh and calf muscle areas were significantly larger in low score males (P < 0.001). High score patients had greater intermuscular fat indices. Regression analysis indicated that vascular stenosis accounted for 5% of the variance in muscle mass, with SFA, PoA, and PTA stenosis having the strongest correlations.

**Discussion:**

Our study reveals how vascular stenosis affects muscle mass and composition in PAD patients, with the SFA, PoA, and PTA having the greatest impact due to their key role in lower limb blood supply. Severe stenosis leads to muscle mass reduction and increased fat infiltration, possibly due to chronic inflammation and oxidative stress. These findings highlight the need to address muscle health in PAD management, as targeting muscle atrophy and fat infiltration could enhance patient outcomes.

**Conclusions:**

PAD severity had a significant effect on the muscles of the lower limbs, especially the stenosis of the SFA, PoA, and PTA. CT evaluation provides a new perspective for understanding muscle loss in patients with PAD.

## Introduction

Peripheral artery disease (PAD) is a globally prevalent atherosclerotic condition[1]. Due to associated risk factors, individuals with PAD are predisposed to cardiovascular and cerebrovascular diseases[1–3]. The prognosis for PAD patients is generally poor, characterized by elevated rates of disability, amputation, and cardiovascular mortality[1, 3].Following lower limb amputation, these patients often experience significant declines in physical, psychological, and social functioning[3, 4].

Sarcopenia is characterized by a decline in both muscle mass and muscle performance[5, 6]. This condition, also referred to as muscle loss, is associated with negative outcomes, including an elevated risk of mortality and increased healthcare costs[7, 8]. Research indicates that the global prevalence of sarcopenia among older adults is approximately 11%[9]. Notably, individuals with cardiovascular disease exhibit higher rates of sarcopenia[10]. A shared pathophysiological mechanism exists between PAD and sarcopenia, wherein PAD can induce changes in the lower limb muscles, thereby impairing their function and metabolic capacity[11].

Current guidelines suggest that the quantification of skeletal muscle is typically conducted using Bioelectrical Impedance Analysis (BIA) and Dual-energy X-ray Absorptiometry (DXA), as well as Computed Tomography (CT)[12, 13]. Among these, CT evaluation of sarcopenia demonstrates a strong correlation with the prognosis of various diseases[14]. Furthermore, CT offers additional metrics over BIA, such as the area of fat infiltration, muscle density, and muscle mass. The integration of these indicators provides a more robust correlation with physical performance[15].

Research has demonstrated that patients with peripheral artery disease (PAD) experience muscle atrophy. Nonetheless, the specificity of this muscular reduction in PAD is not well-defined, and the exact relationship between vascular impairment and muscular alterations remains inadequately understood. To address these gaps in knowledge, we conducted a study to evaluate the area, density, and fat infiltration of both body and lower limb muscles. Our objective was to characterize these muscular changes and investigate their association with vascular stenosis. This approach represents an innovative effort to provide deeper insights into the interplay between vascular and muscular pathologies in PAD.

## Materials and methods

### Study population and data collection

From December 2018 to July 2023, 298 PAD patients who underwent lower extremity computed tomography angiography (CTA) at Beijing Hospital were recruited. Exclusion criteria included: a CTA examination to clinical data collection interval exceeding 3 months, presence of metal implants at the waist, hip, or knee, amputation of lower limbs above or below the knee, and poor CTA image quality. This study received approval from the Research Ethics Committee of Beijing Hospital (approval number:2024BJYYEC-KY071-01), and the requirement for informed consent was waived with authorization.

The clinical characteristics of the patients, along with a review of their medical histories and CTA images, were extracted from the medical records. The data encompassed variables such as age, sex, height, weight, Fontaine stage, and a history of chronic diseases, including coronary artery disease, diabetes, hypertension, as well as smoking habits.

A double lower limb CTA was conducted with a 320-row Aqulion One CT scanner, using 80 mL of iodinated contrast (370 mg/mL) and 40 mL of saline at 4 mL/s. Scanning settings were 320 × 0.5 mm collimation, 120 kV, 80–220 mA, a 512 × 512 matrix, pitch of 1, and an 8–12 s acquisition time.

### Skeletal muscle measurement and vascular assessment

Measurements were taken at four levels: L3, L4, mid-thigh (midpoint between the greater trochanter and femur's medial epicondyle), and the calf's largest soft tissue cross-section[16, 17].

Image segmentation was performed using Slice-O-Matic® software (V4.3),The area (pixels) of each tissue and the average Hounsfield unit (HU) value were recorded. Skeletal muscle area was segmented according to muscle tissue threshold of − 29 to 150HU, and intermuscular fat area was segmented according to adipose tissue threshold of − 190 to − 30HU[18].

The measures include: Skeletal Muscle Area (SMA) at L3; Psoas Muscle Area (PMA) and Psoas Muscle Value (PMV) at L4; Thigh Muscle Area (TMA), Thigh Muscle Value (TMV), and Thigh Intermuscular Fat Area (TFA); Lower-leg Muscle Area (LMA), Lower-leg Muscle Value(LMV), and Lower-leg Intramuscular Fat Area (LFA). Indices are calculated as follows: Skeletal Muscle Index (SMI) = SMA/Height^2^, Psoas Muscle Index (PMI) = PMA/Height^2^, Thigh Intermuscular Fat Index (TFI) = TFA/TMA*100%, Lower-leg Intermuscular Fat Index (LFI) = LFA/LMA*100%.

The degree of arterial stenosis was assessed by identifying the narrowest lesion within the specified arterial segment. The most stenotic cross-sectional image within this segment is selected, and the extent of stenosis is quantified as a proportion of the total luminal diameter. Stenosis is categorized as follows: 0 indicates no stenosis (0–30%), 1 represents mild stenosis (31–50%), 2 denotes moderate stenosis (51–70%), 3 corresponds to severe stenosis (71–99%), and 4 signifies occlusion. The CTA score is calculated by summing the stenosis scores of the following arteries: the Abdominal Aorta (AA), bilateral Common Iliac Arteries (CIA), Internal Iliac Artery (IIA), External Iliac Artery (EIA), Common Femoral Artery (CFA), Superficial Femoral Artery (SFA), Deep Femoral Artery (DFA), Popliteal Artery (PoA), Anterior Tibial Artery (ATA), Peroneal Artery (PeA), and Posterior Tibial Artery (PTA). The maximum possible score is calculated as (2 × 10 + 1) × 4 = 84, with the CTA score ranging from 0 to 84.

To ensure consistent lower limb muscle measurements and vascular lesion scores, a trained graduate student completed all assessments within two weeks, which were then reviewed and adjusted by a experienced attending physicians in vascular intervention. Similarly, all segmentation were done by another student and reviewed by a imaging specialists.

### Statistical analysis

The normally distributed continuous variables were shown as means (± standard deviations). Data that were not normally distributed were expressed as median (quartile spacing).While categorical data are given as numbers and percentages. For continuous variables with a normal distribution, analysis of variance was applied, while the chi-square test was used for categorical variables. For variables that did not follow a normal distribution, the Wilcoxon rank-sum test was applied. Correlations were determined using Pearson's correlation coefficient. Univariate and multiple linear regression analyses were used to examine the link between muscle area and arterial stenosis. The R-squared value assessed the linear model's fit.

Model S1 employed a univariate linear regression approach to examine the relationship between the degree of stenosis in a single artery and the SMI. In contrast, Model S2 utilized a multivariate linear regression framework to explore the relationship between the degree of stenosis across multiple arteries and SMI. Model S3 was further refined by adjusting for potential confounding variables, including age, sex, BMI, smoking status, hypertension, and diabetes. Additionally, Model S3’ was modified to exclude arterial stenosis, allowing for an assessment of the contribution of the arterial stenosis to the overall model fit.

Model T1 used univariate linear regression to examine the link between stenosis in one artery and TMA. Model T2 employed multivariable linear regression to study the association between stenosis in multiple arteries and TMA. Model T3, based on Model T2, adjusted for confounders like age, sex, BMI, HT, smoking, hypertension, and diabetes mellitus. Model T3’ included all factors from Model T3 except for arterial stenosis. Model T4 adjusted Model T2 for SMI to control for factors affecting whole-body muscle. Model T4' was a univariate linear regression of SMI and TMA, adjusted to exclude arterial stenosis, to evaluate its impact on the model fit.

Statistical analysis was conducted using R (Version 4.2.0). A p-value less than 0.05 (p < 0.05) with a 95% confidence interval was considered to be statistically significant.

## Results

A total of 233 patients (177 males, 76%; 56 females, 24%) were included in this study, with a mean age of 68.8 ± 10.0 years. The participants were divided into two groups based on the median CTA-score(36), the high-score group with a CTA-Score > 36 (N = 113) and the low-score group with a CTA-Score ≤ 36 (N = 120). The clinical characteristics of the enrolled patients are shown in Table 1. In the high-score group, 82.3% (93) were male, whereas in the low-score group, 70.0% (84) were male. There was a statistically significant difference in gender distribution between the two groups (p < 0.05). The average age of the high-score group was 70.1 years (± 9.30), and the average age of the low-score group was 67.5 years (± 10.5). There was a significant difference in age between the two groups (p < 0.05). The proportions of smoking, hypertension, and coronary heart disease (CHD) in the two groups (high-score group vs. low-score group) were 24 (21.2%) vs. 20 (16.7%), 47 (41.6%) vs. 40 (33.3%), and 26 (23.0%) vs. 22 (18.3%) respectively. However, these differences did not achieve statistical significance (P > 0.05). Similarly, the distribution of diabetes comparable between the two groups 38 (33.6%)vs.40 (33.3%). And there was no significant difference in BMI 24.3 (± 3.41) vs 23.6 (± 3.30). (P > 0.05for both diabetes and BMI) (Table 1).

**Table 1:** Clinical characteristics of the low-score and high-score groups

	Total *n* = *233*	High *n* = *113*	Low *n* = *120*	P
*Sex*				0.041*
Female	56 (24.0%)	20 (17.7%)	36 (30.0%)	
Male	177 (76%)	93 (82.3%)	84 (70.0%)	
Age (year)	68.8 (± 10.0)	70.1 (± 9.30)	67.5 (± 10.5)	0.049*
BMI (kg/m^2^)	23.9 (± 3.36)	24.3 (± 3.41)	23.6 (± 3.30)	0.145
*Smoking*				0.469
No	189 (81.1%)	89 (78.8%)	100 (83.3%)	
Yes	44 (18.9%)	24 (21.2%)	20 (16.7%)	
*Hypertension*				0.243
No	146 (62.7%)	66 (58.4%)	80 (66.7%)	
Yes	87 (37.3%)	47 (41.6%)	40 (33.3%)	
*Diabetes*				1.000
No	155 (66.5%)	75 (66.4%)	80 (66.7%)	
Yes	78 (33.5%)	38 (33.6%)	40 (33.3%)	
*CHD*				0.472
No	185 (79.4%)	87 (77.0%)	98 (81.7%)	
Yes	48 (20.6%)	26 (23.0%)	22 (18.3%)	
*Fontaine*				0.006*
II	103 (44.2%)	38 (33.6%)	65 (54.2%)	
III	55 (23.6%)	30 (26.5%)	25 (20.8%)	
IV	75 (32.2%)	45 (39.8%)	30 (25.0%)	
CTA score	36.3 (± 14.6)	48.1 (± 8.98)	25.1 (± 8.79)	< 0.001**

BMI body mass index, CHD coronary heart disease

*Statistically significant:**P < 0.001,*P < 0.05

Among males, the mean values of SMA and SMI were higher in the low CTA score group compared to the high score group; however, this difference was not statistically significant (P > 0.05). In females, the trend was reversed, the difference remained statistically insignificant (P > 0.05). Furthermore, the trunk muscle indices PMA, PMI, and PMV exhibited significantly higher values in the low score group than in the high score group among males (P < 0.05). Conversely, these differences were not statistically significant in females (P > 0.05) (Table 2).

**Table 2:** Comparison of Muscle Characteristics in high score Versus low score Gender Subgroups

Parameters	Male				Female		
	Total *n* = *177*	High n = *86*	Low n = *91*		*Total n* = *56*	High* n* = *28*	Low* n* = *28*
SMA (cm^2^)	129 (± 25.5)	125 (± 22.9)	132 (± 27.5)		86.2 (± 16.3)	87.8 (± 18.3)	84.6 (± 14.2)
SMI(cm2 /m2)	43.9 (± 8.35)	42.8 (± 7.84)	45.0 (± 8.72)		34.2 (± 6.03)	34.5 (± 6.67)	33.8 (± 5.42)
PMA(cm2)	22.8 (± 5.33)	21.6 (± 5.32)*	23.8 (± 5.15)*		13.6 (± 3.58)	13.9 (± 3.68)	13.2 (± 3.52)
PMI(cm2 /m2)	7.74 (± 1.80)	7.37 (± 1.86)*	8.09 (± 1.67)*		5.39 (± 1.38)	5.49 (± 1.43)	5.30 (± 1.36)
PMV(Hu)	60.0 (± 9.57)	58.2 (± 10.6)*	61.7 (± 8.21)*		55.6 (± 9.48)	54.6 (± 8.74)	56.6 (± 10.2)
TMA(cm2)	215 (± 51.0)	199 (± 44.8)**	229 (± 52.4)**		148 (± 37.4)	145 (± 41.1)	152 (± 33.6)
TMV(Hu)	51.8 (± 8.58)	49.4(± 9.66**)	54.1(± 6.71)**		47.0 (± 9.43)	45.1(± 8.77)	48.9 (± 9.82)
TFI(%)	8.43(6.15)	10.55(5.60)**	6.77(4.10)**		14.21(11.37)	18.71(11.18)**	8.92(6.76)**
LMA (cm2 /m2)	63.5 (± 14.2)	60.2 (± 13.4)*	66.5 (± 14.4)*		47.3 (± 11.4)	45.5 (± 12.1)	49.2 (± 10.5)
LMV (Hu)	48.4 (± 10.0)	45.2(± 11.4)**	51.4(± 7.42)**		41.0 (± 12.0)	37.7(± 11.8)*	44.3(± 11.4)*
LFI (%)	3.71 (4.56)	4.45( 6.65)*	3.112(3.04)*		5.85(7.92)	8.16(9.07)*	5.15(6.35)*

*SMA* skeletal muscle area, *PMA* psoas muscle area, *PMV* psoas muscle value, *TMA* thigh muscle area, *TMA* thigh muscle value, *LMA* lower-leg muscle area, *LMV* lower-leg muscle value, SMI skeletal muscle index, *PMI* psoas muscle index, *TFI* thigh intermuscular fat index, *LFI* lower-leg intermuscular fat index

*Statistically significant:**P < 0.001,*P < 0.05

In the analysis of thigh muscle indicators, TMA and TMV were significantly elevated in the low score group compared to the high score group among males (P < 0.001), suggesting a reduced overall muscle mass in the high score group. Conversely, this difference was not statistically significant in females (P > 0.05); however, TMV was marginally higher in the high score group than in the low score group (P < 0.05). Regarding calf muscle indices, LMA and LMV were notably higher in the low score group than in the high score group for males (P < 0.001), indicating a diminished localized muscle or body mass in the high score group. In females, the low score group exhibited a significantly higher LMV compared to the high score group (P < 0.05), whereas the difference in LMA was not statistically significant(P > 0.05) (Table 2).

Concerning the intermuscular fat index, in males, the TFI was significantly elevated in the high score group relative to the low score group, and the LFI was similarly higher in the high score group, indicating that the degree of fat infiltration in this population is relatively high. In females, the TFI was also greater in the high score group compared to the low score group; however, this difference was less pronounced than in males. Additionally, in females, the LFI was higher in the high score group, though this difference did not reach statistical significance (Table 2).

**Fig. 1:**
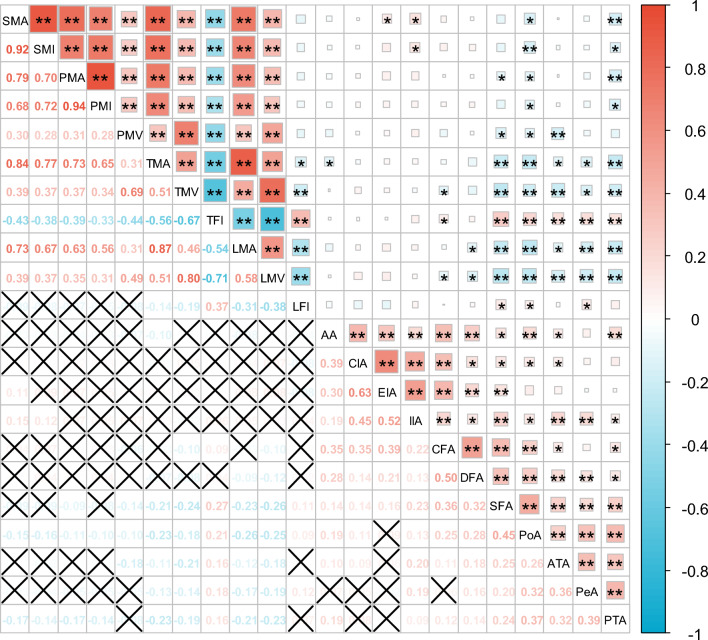
Correlation Heatmap Between Arterial Stenosis Degree and Muscle Parameters. The blue squares represent a negative correlation, and the red squares represent a positive correlation. The numbers within these squares indicate the correlation coefficients. *Statistically significant: **P < 0.001,*P < 0.05

A Pearson correlation analysis of trunk muscle indices revealed a negative correlation between the degree of stenosis in the SFA, PoA and PTA with the SMA, SMI, PMA, PMV and PMI. Notably, the correlations were more pronounced for the PoA and PTA (P < 0.001 or P < 0.05).A Pearson correlation analysis of lower limb muscle indices revealed that the degree of stenosis in the CFA, DFA, SFA, PoA, ATA, PeA and PTA exhibited a negative correlation with TMA, TMV, LMA and LMV. Conversely, a positive correlation was observed with TFI and LFI. Notably, the correlations involving SFA, PoA, ATA, PTA and PeA demonstrated greater statistical significance (P < 0.001 or P < 0.05). Figure 1 illustrates the correlation between these variables.

The linear regression analysis of SMI indicates that, for PoA, the majority of the regression coefficients in Model-s1 and Model-s2 achieved statistical significance (P < 0.05). However, this significance diminished in Model-s3. This suggests that the effect of PoA on the dependent variable is attenuated after adjusting for covariates such as gender, age, and BMI. Regarding PTA, a few coefficients (3) exhibited statistical significance (P < 0.05), yet, upon controlling for additional variables, the PTA coefficients did not significantly impact the dependent variable. (P > 0.05) in the analysis of SFA, the regression coefficients did not achieve statistical significance across all models This suggests that the impact of SFA on the dependent variable may not be substantial (P > 0.05) (Table 3).

**Table 3:** Results of multi-model linear regression for skeletal muscle index (SMI)

Variables	Model S1			Model S2			Model S3	
	β (95%CI)	*P*		β (95%CI)	*P*		β (95%CI)	*P*
*SFA*								
0	0.00 (Reference)			0.00 (Reference)			0.00 (Reference)	
1	− 1.48 (− 4.10 ~ 1.13)	0.267		1.20 (− 1.82 ~ 4.22)	0.436		− 0.40 (− 2.25 ~ 1.46)	0.676
2	− 1.46 (− 4.40 ~ 1.47)	0.330		1.42 (− 1.91 ~ 4.75)	0.403		− 1.32 (− 3.41 ~ 0.76)	0.215
3	− 1.62 (− 4.31 ~ 1.08)	0.240		0.69 (− 2.30 ~ 3.69)	0.650		− 0.50 (− 2.34 ~ 1.34)	0.596
4	− 1.88 (− 4.18 ~ 0.42)	0.109		0.66 (− 2.05 ~ 3.36)	0.634		− 0.58 (− 2.25 ~ 1.09)	0.495
*PoA*								
0	0.00 (Reference)			0.00 (Reference)			0.00 (Reference)	
1	− 3.72 (− 5.93 ~ − 1.51)	0.001		− 3.94 (− 6.52 ~ − 1.36)	0.003		− 1.90 (− 3.50 ~ − 0.31)	0.020
2	− 3.48 (− 6.06 ~ − 0.90)	0.008		− 3.73 (− 6.77 ~ − 0.68)	0.017		− 1.11 (− 3.03 ~ 0.81)	0.256
3	− 3.65 (− 6.76 ~ − 0.55)	0.021		− 3.29 (− 6.70 ~ 0.12)	0.059		− 1.63 (− 3.79 ~ 0.52)	0.138
4	− 4.12 (− 6.52 ~ − 1.71)	< 0.001		− 3.87 (− 6.76 ~ − 0.98)	0.009		− 0.84 (− 2.67 ~ 0.98)	0.365
*PTA*								
0	0.00 (Reference)			0.00 (Reference)			0.00 (Reference)	
1	0.62 (− 1.89 ~ 3.12)	0.631		0.95 (− 1.65 ~ 3.55)	0.476		0.33 (− 1.28 ~ 1.94)	0.685
2	− 0.55 (− 3.66 ~ 2.57)	0.730		0.72 (− 2.61 ~ 4.05)	0.672		1.15 (− 0.92 ~ 3.21)	0.276
3	− 5.78 (− 8.62 ~ − 2.94)	< 0.001		− 4.87 (− 7.88 ~ − 1.87)	0.002		− 1.23 (− 3.13 ~ 0.67)	0.206
4	− 1.66 (− 3.80 ~ 0.49)	0.131		− 0.42 (− 2.79 ~ 1.96)	0.730		0.47 (− 1.01 ~ 1.95)	0.532

Model S1: Univariate linear regression was performed for each of the variables: Superficial Femoral Artery(SFA),Popliteal Artery (PoA), and Posterior Tibial Artery (PTA).

Model S2: Multivariate linear regression included arterial stenosis variables: PoA, SFA, and PTA.

Model S3: Multivariate linear regression was expanded to include additional confounding variables: POA, SFA, PTA, age, sex, BMI, smoking status, hypertension, and diabetes.

Categories 0–4: 0 no stenosis (0–30%); 1 mild stenosis (31–50%); 2 moderate stenosis (51–70%); 3 severe stenosis (71–99%) and 4 occlusion.

In Table 4, both the Multiple R-squared and Adjusted R-squared values for Model-S1 and Model-S2 are low (< 10), suggesting that these models exhibit a poor fit to the data and can only account for a limited degree of variability. Notably, the R-squared value for Model-S2 is lower than the cumulative R-squared values of all variables in Model-S1, indicating potential collinearity among the variables. In contrast, Model-S3’ demonstrates a better fit with an R-squared value of 0.557. However, Model-S3 shows only a marginal improvement in overall fit compared to Model-S3’, implying that the variables PoA, SFA, and PTA have a limited capacity to explain the variability in SMI.

**Table 4:** Comparison of the Goodness-of-Fit of Multiple Linear Regression Models for Predicting Skeletal Muscle Index (SMI)

y	R-Squared	Model-S1			Model-S2	Model-S3’	Model-S3
		SFA	PoA	PTA			
SMI	Multiple R-squared	0.006	0.032	0.046	0.070	0.626	0.648
	Adjusted R-squared	− 0.002	0.0237	0.038	0.045	0.620	0.625

Model S1: Univariate linear regression was performed for each of the variables: Superficial Femoral Artery (SFA), Popliteal Artery (PoA),and Posterior Tibial Artery (PTA)

Model S2: Multivariate linear regression included stenosis variables: PoA, SFA, and PTA

Model S3: Multivariate linear regression was expanded to include additional confounding variables: POA, SFA, PTA, age, sex, BMI, smoking status, hypertension, and diabetes

Model S3': Multivariate linear regression was conducted excluding the arterial stenosis variables, incorporating only the confounding variables: age, sex, BMI, smoking status, hypertension, and diabetes

In Table 5, Model-T1 shows that various PoA values significantly affect the dependent variable (P < 0.05 or P < 0.001). Certain SFA values (1 and 4) and PTA values (1 and 3) also have significant effects (P < 0.001 and P < 0.05, respectively). Only one ATA value is significant, while PeA has low significance.In Model-T2, PoA significantly impacted the dependent variable (P < 0.001 and P < 0.05, respectively), with notable changes in the significance of SFA, PTA, ATA, and PeA, indicating collinearity and PoA's greater representativeness. In Model-T3, SFA and PoA significantly affected the dependent variable (P < 0.001 and P < 0.05, respectively), while PTA and ATA had low significance. In Model-T4, adjusting for SMI altered the significance of all variables, with only a few nearing significance.

**Table 5:** Results of Multi-Model Linear Regression for Thigh Muscle Area (TMA)

Variables	Model T1			Model T2			Model T3			Model T4	
	β (95%CI)	*P*		β (95%CI)	*P*		β (95%CI)	*P*		β (95%CI)	*P*
*SFA*											
0	0.00 (Reference)			0.00 (Reference)			0.00 (Reference)			0.00 (Reference)	
1	− 8.52 (− 16.78 ~ − 0.26)	0.044		1.06 (− 8.40 ~ 10.52)	0.826		− 4.02 (− 10.84 ~ 2.80)	0.249		− 2.28 (− 8.22 ~ 3.65)	0.451
2	− 7.49 (− 16.75 ~ 1.77)	0.114		3.26 (− 7.13 ~ 13.64)	0.539		− 4.22 (− 11.85 ~ 3.40)	0.278		0.07 (− 6.44 ~ 6.59)	0.983
3	− 7.84 (− 16.34 ~ 0.66)	0.071		1.96 (− 7.48 ~ 11.40)	0.684		− 1.83 (− 8.62 ~ 4.96)	0.598		0.00 (− 5.92 ~ 5.92)	1.000
4	− 16.46 (− 23.71 ~ − 9.21)	< 0.001		− 7.27 (− 15.74 ~ 1.21)	0.094		− 10.42(− 16.53 ~ − 4.32)	< 0.001		− 8.52(− 13.83 ~ − 3.20)	0.002
*PoA*											
0	0.00 (Reference)			0.00 (Reference)			0.00 (Reference)			0.00 (Reference)	
1	− 13.14 (− 20.13 ~ − 6.15)	< 0.001		− 10.46(− 18.45 ~ − 2.47)	0.011		− 5.16 (− 10.95 ~ 0.63)	0.081		− 0.53 (− 5.59 ~ 4.53)	0.838
2	− 16.87 (− 25.03 ~ − 8.72)	< 0.001		− 14.59(− 24.07 ~ − 5.11)	0.003		− 6.13 (− 13.13 ~ 0.86)	0.086		− 4.40(− 10.39 ~ 1.60)	0.151
3	− 20.14 (− 29.96 ~ − 10.32)	< 0.001		− 13.88(− 24.62 ~ − 3.13)	0.012		− 9.94 (− 17.86 ~ − 2.02)	0.014		− 5.97(− 12.73 ~ 0.80)	0.084
4	− 18.34 (− 25.95 ~ − 10.74)	< 0.001		− 9.33 (− 18.37 ~ − 0.29)	0.044		− 1.89 (− 8.56 ~ 4.78)	0.579		0.60 (− 5.12 ~ 6.32)	0.837
*PTA*											
0	0.00 (Reference)			0.00 (Reference)			0.00 (Reference)			0.00 (Reference)	
1	4.27 (− 3.68 ~ 12.23)	0.293		2.99 (− 5.77 ~ 11.75)	0.504		1.12 (− 5.17 ~ 7.40)	0.728		2.41 (− 3.08 ~ 7.90)	0.390
2	− 4.86 (− 14.73 ~ 5.02)	0.335		− 5.52 (− 16.67 ~ 5.63)	0.333		− 4.82 (− 12.88 ~ 3.24)	0.242		− 4.09 (− 11.09 ~ 2.90)	0.252
3	− 18.88 (− 27.88 ~ − 9.88)	< 0.001		− 10.46 (− 21.17 ~ 0.26)	0.056		− 4.21 (− 12.10 ~ 3.68)	0.296		− 0.04 (− 6.80 ~ 6.72)	0.991
4	− 11.27 (− 18.07 ~ − 4.48)	0.001		− 7.98 (− 15.85 ~ − 0.10)	0.048		− 4.91 (− 10.61 ~ 0.79)	0.092		− 4.49 (− 9.44 ~ 0.45)	0.076
*ATA*											
0	0.00 (Reference)			0.00 (Reference)			0.00 (Reference)			0.00 (Reference)	
1	− 5.37 (− 15.00 ~ 4.26)	0.275		− 6.07 (− 16.14 ~ 3.99)	0.238		− 0.83 (− 8.24 ~ 6.58)	0.827		− 3.89 (− 10.20 ~ 2.42)	0.228
2	1.51 (− 8.63 ~ 11.65)	0.771		4.38 (− 6.40 ~ 15.16)	0.426		3.73 (− 4.12 ~ 11.58)	0.353		2.21 (− 4.55 ~ 8.98)	0.521
3	− 25.20(− 35.34 ~ − 15.06)	< 0.001		− 17.04(− 28.31 ~ − 5.76)	0.003		− 12.16(− 20.59 ~ − 3.73)	0.005		− 11.72(− 18.80 ~ − 4.64)	0.001
4	− 6.61 (− 14.06 ~ 0.83)	0.082		− 0.78 (− 8.94 ~ 7.37)	0.851		− 1.42 (− 7.39 ~ 4.55)	0.641		− 2.53 (− 7.64 ~ 2.59)	0.333
*PeA*											
0	0.00 (Reference)			0.00 (Reference)			0.00 (Reference)			0.00 (Reference)	
1	8.33 (− 0.06 ~ 16.71)	0.052		10.40 (1.57 ~ 19.22)	0.021		9.77 (3.40 ~ 16.14)	0.003		5.71 (0.16 ~ 11.25)	0.044
2	4.81 (− 3.51 ~ 13.12)	0.258		9.53 (0.64 ~ 18.42)	0.036		9.00 (2.53 ~ 15.48)	0.007		4.34 (− 1.24 ~ 9.93)	0.128
3	− 8.62 (− 17.42 ~ 0.18)	0.056		4.45 (− 5.25 ~ 14.16)	0.369		6.79 (− 0.35 ~ 13.93)	0.063		2.13 (− 3.95 ~ 8.22)	0.492
4	− 4.43 (− 11.27 ~ 2.41)	0.205		5.45 (− 2.15 ~ 13.04)	0.160		5.07 (− 0.42 ~ 10.56)	0.071		0.13 (− 4.64 ~ 4.91)	0.956

Model T1: Univariate linear regression was performed for each of the variables:Superficial Femoral Artery(SFA),Popliteal Artery (PoA),Anterior Tibial Artery (ATA),Peroneal Artery (PeA) and Posterior Tibial Artery (PTA)

Model T2: Multivariate linear regression included arterial stenosis variables: SFA, PoA, PTA, ATA and PeA

Model T3: Multivariate linear regression was expanded to include additional confounding variables: SFA, PoA, PTA, ATA, PeA, age, sex, BMI, smoking status, hypertension, and diabetes

Model T4: Multivariate linear regression was expanded to include SFA, PoA, PTA, ATA, PeA, and SMI

Categories 0–4: 0 no stenosis (0–30%); 1 mild stenosis (31–50%); 2 moderate stenosis (51–70%); 3 severe stenosis (71–99%) and 4 occlusion

In Table 6, the analysis comparing Model-T3 and Model-T3' indicates that vascular variables account for approximately 6% of the model's variance after adjusting for confounding factors. Similarly, the comparison between Model-T4 and Model-T4' demonstrates that vascular variables explain 4% of the model's variance after controlling for potential confounding factors impacting whole-body muscle function. Furthermore, the comparisons between Table 4 and Table 6 suggest that vascular variables have a stronger explanatory power for TMA than for SMI.

**Table 6:** Comparison of the Goodness-of-Fit of Multiple Linear Regression Models for Predicting Thigh Muscle Area (TMA)

y	R-squared	Model-T1					Model-T2	Model-T3’	Model-T3	Model-T4’	Model-T4
		SFA	PoA	PTA	PeA	ATA					
TMA	Multiple R-squared	0.043	0.062	0.073	0.037	0.062	0.168	0.471	0.557	0.612	0.674
	Adjusted R-squared	0.035	0.054	0.065	0.029	0.054	0.130	0.467	0.529	0.611	0.658

Model T1: Univariate linear regression was performed for each of the variables: Superficial Femoral Artery (SFA),Popliteal Artery (PoA),Anterior Tibial Artery (ATA), Peroneal Artery (PeA) and Posterior Tibial Artery (PTA)

Model T2: Multivariate linear regression included arterial stenosis variables: SFA, PoA, PTA, ATA and PeA

Model T3: Multivariate linear regression was expanded to include additional confounding variables: SFA, PoA, PTA, ATA, PeA, age, sex, BMI, smoking status, hypertension, and diabetes

Model T3': Multivariate linear regression was conducted excluding the arterial stenosis variables, incorporating only the confounding variables: age, sex, BMI, smoking status, hypertension, and diabetes

Model t4: Multivariate linear regression was expanded to include SFA, PoA, PTA, ATA, PeA and SMI

Model t4': Univariate linear regression was performed for SMI

## Discussion

In this retrospective study, we examined the relationship between lower extremity vascular stenosis and muscle function. Our findings indicate that the muscles of the lower limbs are significantly impacted by stenosis in the lower limb arteries, whereas the trunk muscles are comparatively less affected. Notably, stenosis in the SFA, PoA, and PTA exhibited more pronounced effects on both lower limb and trunk muscles. Through multiple linear regression modeling, we determined that lower extremity vascular stenosis accounts for approximately 5% of the variance in lower extremity muscle function. This result is novel, highlighting the substantial negative impact of vascular stenosis on lower limb muscles.

Age-related sarcopenia is influenced by intricate interactions among genetic, environmental, and lifestyle factors[19]. The physiological mechanisms underlying sarcopenia are multifaceted, encompassing a range of elements such as diminished regenerative capacity of muscle cells, inadequate nutrient intake, alterations in hormone levels, chronic inflammation, and oxidative stress[20–22].Chronic inflammation and oxidative stress are important mechanisms leading to sarcopenia and are present in PAD. Empirical studies have demonstrated that inflammatory factor levels are frequently elevated in elderly populations, potentially resulting in cellular damage and dysfunction[23, 24].Moreover, increased oxidative stress can impair the structure and function of muscle cells, further accelerating muscle decline[25, 26].

Our study identified that patients with severe lower extremity arterial stenosis exhibited reduced muscle area, decreased Hu values, and increased fatty infiltration, consistent with findings reported in previous studies[15, 27].In patients with PAD, sarcopenia is notably marked by significant muscle wasting in the lower limbs and increased fatty infiltration. Given that the largest muscles in the body are located in the lower limbs, sarcopenia is intricately linked to metabolic processes [28, 29], with metabolic alterations contributing to risk factors for PAD and other atherosclerotic conditions[30, 31]. Within this interconnected framework, the relationship between sarcopenia and the severity of PAD may be bidirectional, collectively resulting in a poor prognosis[32].Furthermore, pathological investigations have demonstrated that the muscle tissue in patients with PAD exhibits chronic inflammation. Additionally, the microcirculation and capillary density within this tissue are markedly reduced compared to healthy controls, indicating ischemia and metabolic dysfunction in the muscles associated with PAD[33].Collectively, these imaging findings may reflect diminished metabolic function and muscle strength in PAD.

We observed that trunk muscles were associated with vascular stenosis, but to a lesser extent especially in the multiple linear regression model. The findings of prior research regarding trunk muscles remain contentious. We posit that trunk muscles are minimally influenced by the blood vessels of the lower limbs, which may lead to the observation of inconclusive or contradictory results due to confounding variables[34, 35]. Nevertheless, existing studies have demonstrated a correlation between metrics such as total body muscle volume and psoas muscle area with adverse outcomes in patients with PAD, including post-revascularization amputation and mortality [30–32]. Consequently, comprehensive assessment of whole-body musculature should not be overlooked.

Existing research has demonstrated a negative correlation between muscle fat infiltration and both gait performance and activities of daily living in patients. This suggests that the extent of muscle fat infiltration may serve as a critical indicator for assessing the functional status of patients with PAD[36].In correlation analysis, it is noteworthy that SFA, PoA, PTA, and other variables demonstrated a higher correlation with lower limb and trunk muscles. This observation may be attributed to the arterial blood supply regions [37], and could also be associated with reduced mobility due to symptoms such as pain[38, 39]. In clinical practice, the SFA and PoA are considered critical vessels, and their recanalization is linked to symptom improvement and prognosis[40, 41]. This indicates that muscle tissue may mediate the adverse effects of PAD in the pathway from vascular stenosis to clinical outcomes. We strongly recommend that muscle assessment be prioritized and incorporated into management objectives in clinical practice. Utilizing the method outlined in this article, obtaining muscle information is straightforward and does not incur additional costs, as CTA for evaluating vascular stenosis is a standard clinical procedure[42]. Treatment strategies for PAD patients should consider improving muscle mass and function, which may include targeted exercise training and nutritional interventions to slow the progression of muscle atrophy and fat infiltration[43].

We employed a comprehensive set of radiologically derived parameters from CTA, including muscle cross-sectional area, muscle density, and intermuscular fat infiltration, to assess muscle characteristics comprehensively. Our analysis firstly revealed a notable correlation between these parameters and arterial stenosis in the lower extremities, particularly verified a specific relationship between the individual vessel and muscle characteristics, which has not been clearly reported in previous studies. The data show that the stenosis of SFA, PoA and PTA exert most biggest effects on muscle mass. Meanwhile, in order to quantify the specific impact of arterial stenosis on the musculature of the lower limbs, we utilized multiple linear regression models and calculated R^2^ values among different models to avoid overestimating the impact of arterial stenosis on muscle properties through correlation analyses. The results show that vascular stenosis in the lower extremities accounted for 5% of the variance in lower extremity muscle mass. This study represents an innovative effort to provide deeper insights into the interplay between vascular and muscular pathologies in PAD.

Our current CTA-score methodology is relatively straightforward, as it does not account for factors such as lesion segment length, calcification, plaque characteristics, and other relevant information. Furthermore, we observed that the initial correlation between the CTA-score and the Fontaine stage was suboptimal. Specifically, a notable proportion of patients with low CTA-scores were classified as Fontaine stage IV, indicating severe symptoms, whereas patients with high CTA-scores were often found to be at Fontaine stage II, indicating milder symptoms. This indicates that merely aggregating the degree of stenosis for each vessel may not constitute the most effective scoring system. We propose the development of prognostic, muscle- and symptom-oriented arterial scoring systems specifically for the lower extremities, which would represent a significant advancement in this field. Achieving this objective necessitates advanced image processing and annotation techniques, as well as the acquisition of a substantial dataset.

Our study did not encompass non-PAD and mild PAD patients, rendering it challenging to determine the onset of muscular impact due to PAD. While the vascular-muscle connection has been identified, the implications of this relationship on the prognosis of PAD patients—encompassing survival, amputation, disability, and mobility—require further investigation. We are conducting an ongoing prospective cohort study to address these limitations.

## Conclusion

PAD severity had a significant effect on the muscles of the lower limbs, especially the stenosis of the SFA, PoA, and PTA. CT evaluation provides a new perspective for understanding muscle loss in patients with PAD.

## Data Availability

No datasets were generated or analysed during the current study.
