# Correction: The correlation between muscle loss and the severity of vascular stenosis in elderly patients with peripheral artery disease: a retrospective analysis utilizing computed tomography

**DOI:** 10.1007/s40520-025-03044-1

**Published:** 2025-05-19

**Authors:** Yanyang Zhang, Wenxin Zhao, Zuoguan Chen, Yixuan Wang, Xihao Zhang, Xue Chang, Yongjun Li, Jihong Yang

**Affiliations:** 1 Department of Geriatrics, Beijing Hospital, National Center of Gerontology, Institute of Geriatric Medicine, Chinese Academy of Medical Sciences & Peking Union Medical College, Beijing, 100005 China; 2 Graduate School of Chinese Academy of Medical Sciences & Peking Union Medical College, Beijing, 100005 China; 3 Department of Vascular Surgery, Beijing Hospital, National Center of Gerontology, Institute of Geriatric Medicine, Chinese Academy of Medical Sciences & Peking Union Medical College, Beijing, 100005 China; 4 Department of Imaging, Beijing Hospital, National Center of Gerontology, Institute of Geriatric Medicine, Chinese Academy of Medical Sciences & Peking Union Medical College, Beijing, 100005 China; 5 Department of Geriatric, Beijing United Family Hospital, Beijing, 100015 China

**Aging Clinical and Experimental Research (2025) 37:78**

10.1007/s40520-025-02996-8

In this article the author’s name Yanyang Zhang was incorrectly written as Yangyang Zhang.

The original article has been corrected.

